# Serum Metabolomics Identifies Altered Bioenergetics, Signaling Cascades in Parallel with Exposome Markers in Crohn’s Disease

**DOI:** 10.3390/molecules24030449

**Published:** 2019-01-27

**Authors:** Yunjia Lai, Jingchuan Xue, Chih-Wei Liu, Bei Gao, Liang Chi, Pengcheng Tu, Kun Lu, Hongyu Ru

**Affiliations:** 1 Department of Environmental Sciences and Engineering, Gillings School of Global Public Health, CB #7431, University of North Carolina at Chapel Hill, Chapel Hill, NC 27599, USA; lai7@live.unc.edu (Y.L.); xuejc@email.unc.edu (J.X.); ericleah@email.unc.edu (C.-W.L.); liang16@live.unc.edu (L.C.); ptu@live.unc.edu (P.T.); kunlu@unc.edu (K.L.); 2 NIH West Coast Metabolomics Center, University of California at Davis, Davis, CA 95616, USA; beigao@ucdavis.edu; 3 Department of Population Health and Pathobiology, North Carolina State University, Raleigh, NC 27695, USA

**Keywords:** Inflammatory Bowel Disease, Crohn’s Disease, Serum Metabolomics, docosahexaenoic acid, Tryptophan Metabolism, ergothioneine, gut microbiota, Exposome

## Abstract

Inflammatory bowel disease (IBD) has stimulated much interest due to its surging incidences and health impacts in the U.S. and worldwide. However, the exact cause of IBD remains incompletely understood, and biomarker is lacking towards early diagnostics and effective therapy assessment. To tackle these, the emerging high-resolution mass spectrometry (HRMS)-based metabolomics shows promise. Here, we conducted a pilot untargeted LC/MS metabolomic profiling in Crohn’s disease, for which serum samples of both active and inactive cases were collected, extracted, and profiled by a state-of-the-art compound identification workflow. Results show a distinct metabolic profile of Crohn’s from control, with most metabolites downregulated. The identified compounds are structurally diverse, pointing to important pathway perturbations ranging from energy metabolism (e.g., β-oxidation of fatty acids) to signaling cascades of lipids (e.g., DHA) and amino acid (e.g., L-tryptophan). Importantly, an integral role of gut microbiota in the pathogenesis of Crohn’s disease is highlighted. Xenobiotics and their biotransformants were widely detected, calling for massive exposomic profiling for future cohort studies as such. This study endorses the analytical capacity of untargeted metabolomics for biomarker development, cohort stratification, and mechanistic interpretation; the findings might be valuable for advancing biomarker research and etiologic inquiry in IBD.

## 1. Introduction

The inflammatory bowel diseases (IBD) Crohn’s disease and ulcerative colitis (UC) are common, relapsing, and inflammatory disorders of the gastrointestinal (GI) tract, resulting in abdominal pain, diarrhea, internal bleeding, and many other complications [1,2]. Among the subtypes, Crohn’s disease can impact any part of the GI tract, starting often with small intestine, whereas UC’s damage is limited to colon and rectum. Over the past two decades, IBD has stimulated much interest among gastroenterologists, geneticists, and immunologists because of its surging incidence and the associated health impacts in the U.S. and worldwide [3,4]. The exact cause of IBD, however, remains incompletely understood, though it is accepted that genetics, the immune system, and environmental factors all play a role [5,6,7]. To date, genome-wide association studies (GWAS) have identified more than 200 associated genes [8]. Each individual mutation adds to the risk and degree of inflammation, mainly via their impacts on the immune system. Importantly, evidence from in vivo and in vitro models supports that a dysregulated immune response against commensal intestinal microbiota is responsible for the onset of IBD, particularly in genetically susceptible individuals [9,10,11]. There are also dominant environmental components to the pathogenesis of IBD as well [7]; one notable and most thoroughly documented example is smoking, which seems interestingly favorable in UC subjects, but exacerbates the progression of Crohn’s [12].

Despite much efforts stated, knowledge gaps persist towards a complete understanding of IBD pathogenesis. It is still challenging to achieve early diagnostics, and currently, there is no cure for IBD. Biomarker is surprisingly lacking for stratifying IBD subjects in terms of, for example, disease status (active vs. inactive) and subtypes (Crohn’s vs. UC) in a fast and precise manner [13]. For mechanism that drives the onset of IBD, the role of gut microbiota is not clearly delineated, and many unknown environmental factors need to be detected and defined. To tackle all these, the emerging metabolomics approach has started to show promise [14]. Existing metabolomic profiling, mostly nuclear magnetic resonance (NMR) spectroscopy-based, has been performed on a range of IBD sample matrices, including serum [15], urine [16] and feces [17]. However, these studies encounter analytical limitations like low sensitivity and low compound annotation rates. By contrast, mass spectrometry (MS) is sensitive, specific and versatile for resolving a wide range of structures in a high-throughput fashion [18]. As MS continues to evolve, some MS-based metabolomic studies, targeted or untargeted, started reporting novel findings for advancing IBD research. For example, a recent metabolomic study on a pediatric cohort with Crohn’s and UC describes metabolic features in serum and feces that may differentiate Crohn’s disease from UC for adolescent individuals [19]. Another study used targeted serum metabolomics and identified lipid metabolism and amino acid metabolism as key avenues to distinguish between Crohn’s disease and UC [20]. Yet, some results are debatable and deserve validation as well as in-depth discussion mechanism-wise. Of note in these metabolomics studies, little has been reported on environmental factors that might impact the IBD outcomes.

The main goal of this study is to conduct a MS-based untargeted metabolomic analysis for an adult human cohort of Crohn’s disease, for which both active and inactive cases are included. We hypothesize that metabolomics can not only distinguish between the diseased and healthy controls, but confers distinct metabolic patterns among active and inactive states (i.e., in remission). Herein, our specific aims are to detect important metabolic changes, identify biomarkers, and discover exposures or novel metabolites that bear specificity to Crohn’s cohorts. This study may assist our understanding of Crohn’s disease and benefit future biomarker development and exposomic profiling for tackling intestinal inflammation in general.

## 2. Results

### 2.1. Global Metabolomic Profile of the Crohn’s Disease

**Figure 1:**
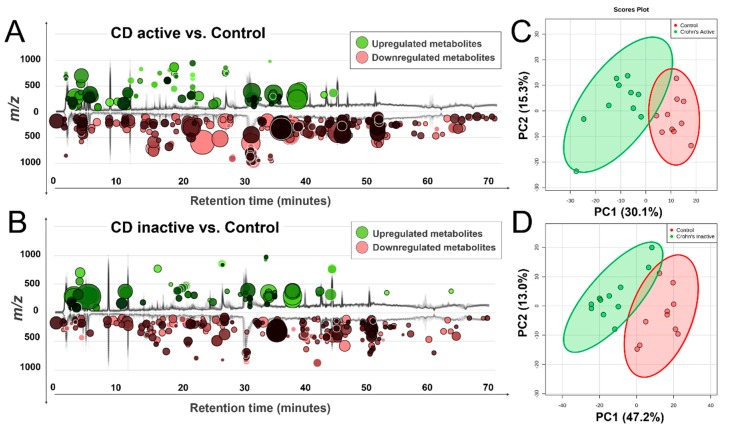
Distinct metabolic profiles of Crohn’s disease compared with control, as revealed by total ion chromatogram (TIC)-based metabolomic cloudplots defined by Welch’s *t*-test (*p* < 0.05) (Panel **A**, **C**) and principal component analysis (PCA) plots with 95% confidence area highlighted (Panel **B**, **D**), which passed permutation tests as part of the validation procedures.

Figure 1 illustrates the global view of Crohn’s metabolic characteristics. Welch’s *t*-test (Figure 1A,C) reveals a number of molecular features that were markedly changed in the Crohn’s, regardless of the disease status (active or inactive). Despite fold change differences, consistency in feature overlapping was seen between active and inactive Crohn’s, with most metabolites downregulated and the active cases embracing a more pronounced scale of change. Principal component analysis (PCA) complies with the observation, and metabolomes of Crohn’s were well separated from the control (Figure 1B,D).

To identify metabolites of interest, four dimensions of analytical data, including accurate mass, isotopic ratios, MS/MS spectra, and chromatographic retention time were used. In total, MS-FINDER 2.30 successfully resolved 124 compounds (Table S1) of diverse structures and functions, ranging from amino acids, lipids, nucleosides, to novel exogenous bio-transformants. This structural diversity endorses the analytical capacity of untargeted metabolomics as a guiding tool for etiologic inquiry for Crohn’s, but meanwhile, it highlights a challenge to resolving the metabolic complexity of pathological changes pertaining to intestinal inflammation.

### 2.2. Integrated Pathway Analysis

**Figure 2:**
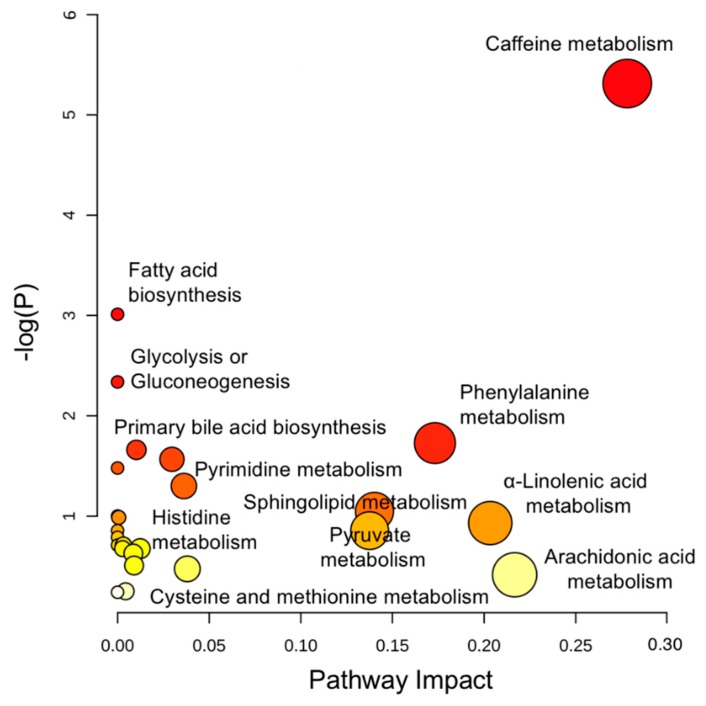
Perturbed pathways identified in MetaboAnalyst 3.0 using hypergeometric test for over-representation analysis and relative-betweenness centrality for pathway topology analysis. Here, the *x*-axis marks the pathway impact and the *y*-axis represents the pathway enrichment. Each node marks a pathway, with larger sizes and darker colors representing higher pathway enrichment and higher pathway impact values.

Integrated pathway analysis was performed in MetaboAnalyst 3.0 (Montreal, QC, Canada) to map all annotated metabolites into biochemical pathways for advancing mechanistic interpretation. As Figure 2 shows, multiple pathways were perturbed, ranging from endogenous metabolism (e.g., amino acids, lipid catabolism) to biotransformation of xenobiotic exposures (e.g., caffeine) with an impact value of 0.01–0.3. Specifically, altered metabolism of amino acids, such as tryptophan (Trp) and phenylalanine (Phe), points to important signaling cascades where microbes might play a role. Also, bioenergetic status was markedly changed in the Crohn’s, as reflected in the alteration of fatty acid oxidation, pyruvate metabolism, and glycolysis. Further, extensive detection of xenobiotics (e.g., caffeine) in blood highlights the ubiquity of environmental exposures, calling on future human studies to stratify cohorts while adjusting for environmental risk factors. To better assist in-depth pathway analysis, a MetaMapp view (Figure S2) was constructed to visualize individual metabolites in network clusters by both biochemical links (KEGG) and chemical similarity (Tanimoto score > 0.7).

### 2.3. Enhanced β-Oxidation of Fatty Acids and Altered Lipid Cascades

**Figure 3:**
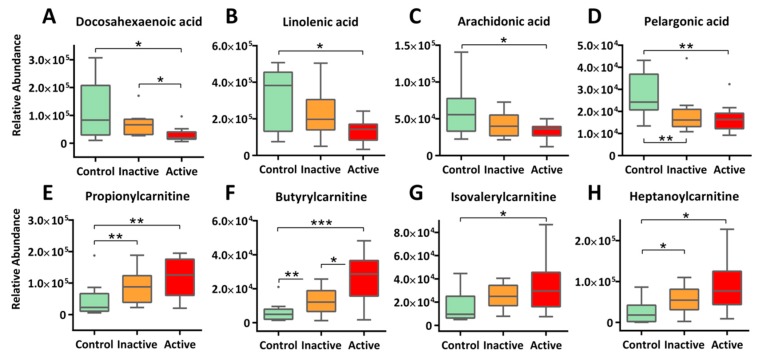
Enhanced β-oxidation of fatty acids and altered lipid signaling cascades is observed in Crohn’s patients, as evidenced by decreasing long-chain fatty acids (**A–C**) and medium-chain fatty acids (**D**), as well as increasing acylcarnitine (**E–H**) in blood circulation. The *y*-axis measures the peak height of the profiled metabolites, and the *x*-axis marks the three IBD groups for comparison, namely control (green), inactive (orange), and active (red), outliers, represented as dots, were excluded from comparison. *** *p* < 0.001, ** *p* < 0.01, * *p* < 0.05.

Striking bioenergetic changes were observed in both active and inactive Crohn’s, as characterized by enhanced fatty acid catabolism. As illustrated in Figure 3, long-chain fatty acids, such as docosahexaenoic acid (DHA, Panel A, −4.4 fold), linolenic acid (Panel B, −3.02 fold), and arachidonic acid (Panel C, −2.05 fold), were markedly decreased in the serum of active Crohn’s patients, so were medium-chain fatty acids like pelargonic acid (Panel D) and caprylic acid (Table S1). Conversely, a range of acylcarnitines, e.g., propionylcarnitine, butyrylcarnitine, isovalerylcarnitine, and heptanoylcarnitine (Figure 3E–H), were largely upregulated in active Crohn’s with a fold change of 2.24–4.19. Inactive Crohn’s patients followed the same trend of reduced fatty acids as well as increased acylcarnitines, though at a small scale of change. It is well established that carnitine shuttle constitutes the rate-limiting step for mitochondrial β-oxidation of fatty acids, especially for those with 14 or more carbons [21]. Here, depleted fatty acids coupling with upregulated acylcarnitines together holds strong evidence that Crohn’s patients encounter a heightened energy demand that is accompanied with pathogenetic progression. From a functional perspective, a decline in fatty acids may also indicate altered lipid cascades in response to inflammation and immune regulation under the state of disease. Notable examples include DHA (Figure 3A) and sphingosine 1-phosphate (Table S1), both of which are shown in emerging studies to play essential roles in modulating inflammatory responses.

### 2.4. Altered Amino Acid Metabolism

**Figure 4:**
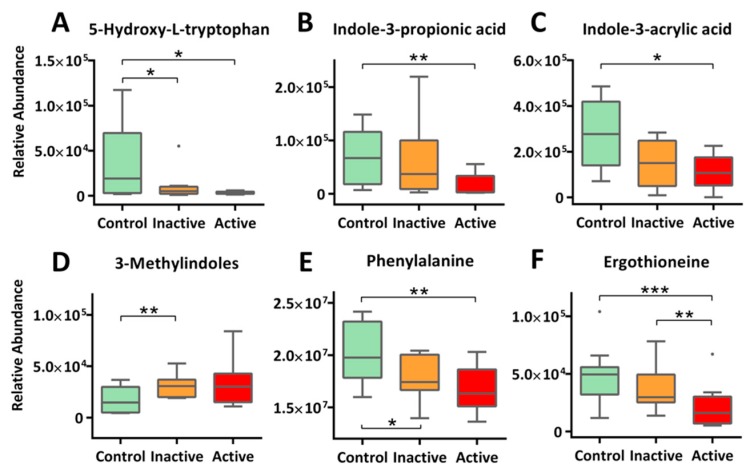
Altered metabolism of amino acids were extensively observed for Crohn’s patients. This includes diminishing 5-HTP, the immediate precursor of 5-HT (**A**), microbe-mediated tryptophan metabolism (**B**–**D**), phenylalanine metabolism (**E**), and histidine metabolism (**F**). The *y*-axis measures the peak height of the profiled metabolites, and the *x*-axis marks the three IBD groups for comparison, namely control (green), inactive (orange) and active (red), outliers, represented as dots, were excluded from comparison. *** *p* < 0.001, ** *p* < 0.01, * *p* < 0.05.

Our metabolomic profiling also identified perturbations in multiple amino acid metabolism in the Crohn’s. Important findings include Trp metabolism (Figure 4A–D), Phe metabolism (Figure 4E), and His metabolism (Figure 4F). First and foremost, we detected 5-hydroxyl-L-tryptophan (5-HTP), the immediate precursor of neurotransmitter 5-hydroxytryptamine (5-HT, or serotonin), at much reduced levels in the serum of Crohn’s patients: 11.9-fold decrease for active and 3.6-fold for inactive, respectively (Figure 4A). Reduced circulating 5-HTP may indicate a diminished pool of 5-hydroxytryptamine (5-HT, or serotonin) in the brain as well as perturbed 5-HT pathways in the gut, which were reported to be associated respectively with psychological comorbidities (e.g., depression) and altered intestinal permeability (with mucosal inflammation) [22,23,24]. For Trp metabolism, we also detected a range of indolic derivatives of microbes in Crohn’s patients. These include indole-3-propionic acid (IPA, −3.66 fold, active), indole-3-acrylic acid (IAcrA, −2.85 fold, active), and 3-methylindole (skatole, +2.22 fold, inactive). Previous studies have identified these indole-containing compounds as potent ligands of important transcription factors such as the aryl hydrogen receptor (AhR), a major xenobiotic metabolic mediator, and more importantly, a critical regulator of immunity and inflammation [25,26,27]. Thus, a role of gut microbes in regulating intestinal inflammation in the context of IBD is highlighted 

Phe is another essential aromatic amino acid that modulates intestinal inflammation. Our study detected significantly lower levels of circulating Phe in Crohn’s, with a trend of active < inactive < control (Figure 4E). Phe has been reported to possess protective effects in the treatment of inflammatory bowel disease via inhibiting tumor necrosis factor-α (TNF-α) productions while enhancing immune responses [28]. A recent report validated the ameliorative effects of Phe (in the form of chromium complex) on indomethacin-induced IBD in rats and suggested that the benefits comes from the anti-oxidant and anti-inflammatory nature of Phe [29].

We also detected much reduced levels of circulating ergothioneine (ET) for Crohn’s. As shown in Figure 4F, serum ET levels not only resolved active Crohn’s (−2.3 fold) from the control but separated inactive cases from control (−1.3 fold) as well. Consistencies of results are found in multiple previous cohort studies [30,31]. ET naturally occurs as a histidine metabolite converted by fungi or mycobacteria but can be obtained from diet such as mushrooms and meat. Importantly, ET exhibits protective effects, functioning both as an antioxidant and a neuroprotective agent. Depletion of ET has been shown to make mammalian cells more sensitive to oxidative stress, resulting in lipid peroxidation and mitochondrial DNA damages, which might be responsible for the exacerbated condition of IBD [32].

### 2.5. Xenobiotics and Novel Metabolites: A Call for Exposomics

**Figure 5:**
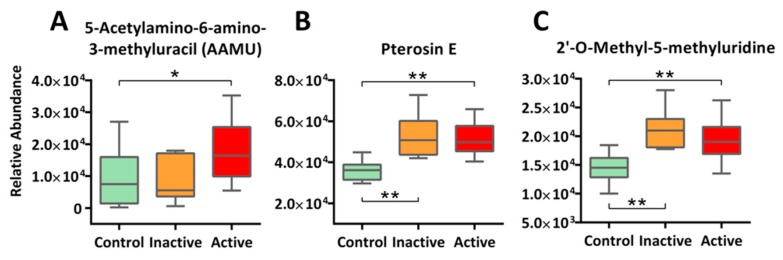
Other than canonical pathways, xenobiotics and novel metabolites were detected in our study at stratified levels among active Crohn’s, inactive Crohn’s, and the Control. Examples include xenobiotic marker AAMU (Panel **A**) and Pterosin E (Panel **B**), as well as novel metabolite 2′-*O*-methyl-5-methyluridine (Panel **C**). The *y*-axis measures the peak height of the profiled metabolites, whereas the *x*-axis marks the three IBD groups for comparison, namely control (green), inactive (orange), and active (red), outliers, represented as dots in the boxplot, were excluded from comparison. ** *p* < 0.01, * *p* < 0.05.

Interestingly, alongside canonical metabolic pathways, we detected a constellation of xenobiotic chemicals and their metabolites, with many at stratified circulating levels among active Crohn’s, inactive Crohn’s, and control. One example to note is 5-acetylamino-6-amino-3-methyluracil (AAMU), a major caffeine metabolite [33] that is detected at significantly higher levels in the sera of active Crohn’s patients (+1.9 fold) as compared with the control group (Figure 5A); further interpretation is provided in the Discussion Section. We also found other xenobiotics, ranging from food biomarkers (e.g., Pterosin E from root vegetables, S-allylcysteine from garlic, and methyl cinnamate from spices), drugs (e.g., simvastatin, atenolol), to biotransformants of environmental pollutants (e.g., monoethylhexyl phthalic acid, MEHP) in most if not all our subjects. Together, our results highlight the ubiquitous, complex, and erratic nature of environmental exposures, though what caused the differentiated levels as well as their etiologic roles in IBD remain a mystery. Indeed, the field of exposomics is only at its budding stages in epidemiological investigation, and much needs to be done to enable sensitive, comprehensive and real-time exposure characterization for unravelling causal factors of IBD and of other complex chronic diseases [34,35]. Aside from exogenous exposure, some novel endogenous metabolites were detected as well. One such metabolite is 2′-*O*-methyl-5-methyluridine, a nucleoside derivative that has never been reported in biological samples before, but was found in our study, with upregulated levels in both active and inactive cases of Crohn’s (Figure 5C). Previous studies suggested that this compound can be endogenously derived via 2′-*O*-methylation of 5-methyluridine that is likely a result of DNA methylation, a common epigenetic modification scheme [36]; the whole process is involved in codon recognition by *t*RNA [37]. Future studies that link this compound to IBD are warranted.

## 3. Discussion

In this study, we conducted a serum metabolomics investigation on an adult cohort of Crohn’s disease, for which both active and inactive states were examined. Regardless of disease status, our metabolomic data successfully resolved a distinct metabolic pattern of Crohn’s from healthy controls, with most metabolites downregulated. Multivariate statistics coupling to downstream structural identification procedures unravels a diverse collection of metabolites to explore, ranging from amino acids, lipids, nucleotides, to xenobiotic biotransformants, etc. Integrated pathway analysis further identifies metabolic pathways in response to Crohn’s, spanning bioenergetics (e.g., β-oxidation of fatty acids), signaling pathways (e.g., DHA, Tryptophan), biotransformation of xenobiotics (e.g., caffeine), as well as novel metabolites (e.g., 2′-*O*-methyl-5-methyluridine). Overall, our findings endorse the analytical capacity of LC-MS metabolomics for etiologic research as such, and might inform future studies on intestinal inflammation, specifically regarding regulatory roles of gut microbiota and the impact of environmental exposures.

First, altered energy metabolism is involved in Crohn’s pathogenesis. Here, enhanced mitochondrial β-oxidation, characterized by diminished fatty acids and upregulated acylcarnitines, suggests high energy demands on Crohn’s body recruiting immune cells to combat the inflammatory status; individuals with active Crohn’s disease undergo larger energetic change than the inactive ones. Under such surging energy demands, inflammatory hypoxia is likely to occur and further exacerbates immune responses [38]. Interestingly, a recent IBD metabolomics study reports decreased fatty acid oxidation for Crohn’s, with reductions in both fatty acids and acylcarnitines in blood; no mechanistic explanation has been offered [20]. Future studies are needed to address this conflict for mechanistic elucidation. Other than bioenergetics, altered lipid signaling cascades are suggested [39]. For example, multiple studies have questioned if DHA, a long-chain ω-3 polyunsaturated fatty acid, plays certain role in ameliorating the inflammatory responses in IBD [40,41]. Using interleukin (IL)-10 knockout mice as an IBD model, a recent study observed high levels of DHA in plasma and large intestine and concluded that lipid mediators, such as DHA and arachidonic acid, may contribute to the disease course of IBD [39]. Another case of interest is the downregulation of sphingosine 1-phosphate (S1P, d16:1) in active Crohn’s patients as compared to the control counterparts (Table S1). S1P (d16:1) is a derivative of S1P, with the latter playing critical regulatory roles in various physiological and pathogenetic contexts, and more recently, in inflammatory responses [42]. Downregulated S1P (d16:1) might indicate a shortage of S1P. The result is consistent with a recently published case-control pilot study [43], which determined the whole sphingolipid profiles and S1P-related gene expression in the colon tissues of IBD patients. The results identified an association between elevated transcriptomic signatures and active IBD status, which were normalized in remission cases. The authors also observed high levels of pro-apoptotic and pro-inflammatory sphingolipids.

Mounting evidence suggests that intestinal microbiota play a pivotal role in maintaining the health of the bowel [44,45]. Dysbiosis of gut microbes has been shown to induce unsolicited immune responses, destroy the integrity of intestinal epithelial barrier (IEB), and spark systemic inflammation, resulting in diseases spanning metabolic syndrome [46], autoimmune diseases [47], and cancer [48]. In this study, we detected for Crohn’s patients an array of altered indole-containing metabolites that were endogenously converted by gut bacteria from approximately 4%–6% of the total tryptophan supply [23]. As the enzymatic processing is species-dependent and influenced by interspecific interactions, altered levels of indole acid derivatives may help assess a dysbiotic status and delineate bacteria-mediated immune responses. For example, we detected a significantly lower level of circulating IPA in active Crohn’s patients (−3.66 fold); this may be attributed to reduced abundance of *Clostridium sporogenes* or other species under the order of Clostridiales which exclusively produce IPA in the gut [49]. As evidenced in a DSS-induced colitis model, IPA protects lipopolysaccharide (LPS)-challenged mice by activating AhR to promote interleukin-10 (IL-10, an anti-inflammatory cytokine) production while suppressing gene expression of TNF-α, a proinflammatory cytokine [50]. A lack of IPA thus suggests a dysbiotic status in Crohn’s cohorts likely characterized by a diminished Clostridiales community. Similarly, reduced IAcrA levels also indicate a dysbiotic gut with impaired IEBs in Crohn’s. Like IPA, IAcrA binds to AhR and exerts an anti-inflammatory effect. Moreover, IAcrA protects IEBs through targeting the pregnane X receptor (PXR) for promoting of mucin 2 (*Muc2*) gene expression [51]. A recent paper has identified a mucin utilizer *Peptostreptococcus russellii* alongside several other Clostridiales candidates as IAcrA producers [52]. Through fecal metagenomic analysis, the authors discovered for IBD patients a shrinking genetic capacity of microbes to use mucins and metabolize Trp. In situ supplementation (through fecal microbiota transplant, FMT, for example) of IPA, IAcrA, or their microbial producers at sites of inflammation in the bowel may be a cure for Crohn’s patients. Skatole is another gut bacteria-derived Trp metabolite but often in low and shifting abundances [53]; the function of this compound has recently been well summarized by Gao et al. [23]. In this study, we detected higher levels of skatole in inactive Crohn’s compared with the control. Previous studies identified *Clostridium* and *Lactobacillus,* etc., as capable to decarboxylate indole-3-acetic acid (IAA) into skatole, in both small intestine and colon of swine models [54,55]. Importantly, skatole can selectively inhibit the growth of certain gram-negative bacterial species, such as *Lactobacillus acidophilus*; higher fecal skatole levels were also found in patients who suffer from disturbed intestinal digestion [53]. To conclude, upregulated skatole may further reflect a dysbiotic gut and compromised health status for Crohn’s, although the underlying regulatory mechanism remains to be defined.

As part of the Trp metabolism, we also detected massive reduction of circulating 5-HTP in Crohn’s patients. 5-HTP is the immediate precursor for the synthesis of 5-HT, a brain neurotransmitter and a regulatory factor in the GI tract [56]. Since intestinal enterochromaffin cells (ECs)-derived 5-HT (accounting for 90% in human body) does not cross blood-brain barrier (BBB), the amount of 5-HT in the brain solely relies on in situ central nervous system (CNS) synthesis by serotonergic neurons [57]. Circulating 5-HTP, however, can easily cross BBB and thus controls the availability of 5-HT in the brain [58]. A shrinking pool of circulating 5-HTP in our Crohn’s cohorts is highly likely to result in a lack of 5-HT in the brain and impact serotonergic neurotransmission for emotional processing. This may explain for depression, anxiety and many other comorbid mental symptoms reported in Crohn’s cases [24]. With high sensitivity, diminishing blood 5-HTP levels may help predict psycho-related comorbidities with Crohn’s disease. In addition, depleted 5-HTP in blood may also point to altered 5-HT pathways in the gut, where microbes are heavily involved. A recent landmark study on the metabolism of gut-derived 5-HT delineates how spore-forming bacteria (Sp) regulates host peripheral 5-HT levels, with profound effects on host physiology, gut motility, and hemostasis [22]. Importantly, the authors observed increased levels of colonic and blood 5-HT in germ-free (GF) mice in response to elevating luminal metabolites of Sp, likely by signaling directly to colonic ECs for promoting tryptophan hydrolase 1 (Tph1) expression and 5-HT synthesis. Future studies are warranted, to gain a quantitative understanding of the role of gut microbiome in IBD pathogenesis, for instance, through long-term monitoring of a more complete list of Trp metabolites in IBD patients.

Remarkably, we detected ET at much reduced levels in Crohn’s serum, with active and inactive cases well stratified. As a fungal metabolite of His, ET is typically obtained from food and maintains at levels of a small range among healthy human populations [59]. Previous studies imply an essential role of ET in suppressing intestinal inflammation while possessing protective effects on IBD individuals. For instance, ET seems to exert anti-inflammatory effects that protect mammalian cells under the challenge of oxidative stress [32]. On the other hand, loss of ET, due to for example, mutation or knockout of *octn1* gene encoding organic cation/carnitine transporter 1 (OCTN1) that exclusively transports ET across intestinal epithelia, is associated with increased risks of inflammatory diseases such as rheumatoid arthritis (RA) and Crohn’s disease [59,60]. As OCTN1 is solely expressed in small intestine, reduced ET in blood may help distinguish between Crohn’s and UC, as the latter mainly affects large intestine [2]. Although mechanistic understanding is lacking for explaining ET reduction in Crohn’s, recent efforts have been made, for instance, on testing if immune cells (e.g., monocytic macrophages) are partially involved, likely by trapping ET in inflammatory intestinal tissues [59]. Taken together, ET shows promise for serving as a biomarker specifically for Crohn’s disease, and may further aid in medical efficacy assessment by differentiating an inactive status from active counterparts.

Environmental exposures represent a vital component that drives the IBD outcomes. In this study, we detected multiple xenobiotics (and their metabolites) for Crohn’s and surprisingly, many are found at stratified serum levels among active Crohn’s, inactive Crohn’s and Control, ranging from food biomarkers, drugs, to environmental pollutants. However, how they entered the human body and to what extent they exert health effects pertaining to the onset and progression of IBD remains poorly characterized. One notable example is coffee consumption, for which our study detected a higher level of AAMU, a major caffeine metabolite, within the individuals with active Crohn’s disease. Current studies linking caffeine intake with IBD are still lacking, despite the popularity of caffeine worldwide. Controversies remain on the distribution of caffeine intake as well as its potential role in the pathogenesis of IBD. For instance, the existing dietary guidelines for IBD recommends avoiding caffeine to combat IBD based on existing data [61]. An early dietary analysis discovered anti-thiamin properties of caffeine and associated decaffeinated coffee with a better clinical state of UC compared with caffeine-containing group [62]. A recent prospective cohort study compared Eastern and Western European individuals with IBD; results showed that caffeine intake was similar across both geographical locations for both IBD types, and associated high caffeine intake with a severe disease course with Crohn’s follow-ups [63]. Another study surveyed the thoughts of IBD patients on the impact of coffee consumption, and overall concluded a negative perception, which is more often attributed to coffee in patients with Crohn’s disease [64]. Counterintuitively, an experimental trial employing a dextran sulfate sodium (DSS)-induced colitis murine model shows that oral intake of caffeine is safe and even protective in the development of acute colitis; the benefit is likely resulted from the inhibition of Chitinase 3-like 1 (CHI3L1) in response to proinflammatory cytokines [65]. For this study, it would be of value to unravel the factors underlying such heightened levels of AAMU as well as the associated health impacts on the Crohn’s subjects. The increase is likely due to elevated intake of caffeine, a compromise in their metabolizing capacity, and a leaky gut in the diseased that allow intestinal caffeine metabolites to come across and reach the circulation system [66]. Overall, the common detection of xenobiotics raises the open question as to whether this difference is caused by distinct uptake kinetics of exposures (e.g., as affected by occupation, diet, and lifestyle etc.) or altered metabolizing capacities of them that are specific to Crohn’s progression. Future studies are highly needed to screen and identify unknown environmental risk factors, and to address their etiologic contributions.

The strength of this study lies in the use of untargeted metabolomic profiling, a state-of-the-art compound identification strategy, and the inclusion of inactive Crohn’s subjects. These allow a more comprehensive and stratified view of important metabolic changes as well as the discovery of novel markers/compounds specific to Crohn’s disease in different states. Despite the many informative findings, future studies are needed to validate our findings in expanding cohorts or through correlation analysis with histological data. Likewise, our metabolomics profiling is only cross-sectional and can only serve as a guide rather than direct causal interpretation. In this sense, untargeted metabolomics coupling to a longitudinal study design is desired in future IBD studies to delineate pathogenic causes while adjusting for the many complex environmental confounders.

## 4. Materials and Methods

### 4.1. Characteristics of Participants

Human serum samples were collected by the Massachusetts General Hospital Crohn’s and Colitis Center with written and informed consent obtained and was approved by the MGH Institutional Review Board. All samples were anonymized prior to the receipt at the Massachusetts Institute of Technology (MIT) and approved for use by the MIT Committee on the Use of Humans as Experimental Subjects Committee (COUHES). Human serum samples were obtained from patients that were diagnosed to be either clinically active or in remission for CD (n = 10 for each group) using the Simple Clinical Colitis Activity Index (SCCAI; active defined as > 4; inactive ≤ 4) or the Harvey-Bradshaw Index (HBI; active defined as ≥ 5; inactive defined as < 5), respectively. Additionally, non-IBD serum was obtained from healthy human donors (Control). Each serum sample was associated with a clinically assessed disease score at the time of sample collection (control: 0; CD active: 12.9 ± 3.6; CD inactive: 0). There was no statistically significant difference in the age distribution (control: 52.1 ± 12.8; CD active: 48.9 ± 17.8; and CD inactive: 39.5 ± 18.2) and gender of the groups. Moreover, all CD active patients are treated with medication, while 40% CD inactive patients are not on any medication. A summary of the clinical data can be found in Table S3.

### 4.2. Sample Preparation

Metabolites were extracted by adding 180 µL cold methanol to 20 µL serum. Then, all samples were vortexed for 1 min, incubated at 4 °C for 20 min, and centrifuged at 12,000 rpm for 20 min. The supernatant was collected, dried in a SpeedVac evaporator (Thermo Fisher Scientific, Waltham, MA, USA), and resuspended in 30 µL of 98:2 water/acetonitrile upon instrumental analysis.

### 4.3. Metabolomic Profiling by LC-MS

Metabolomic analysis was performed on a LC/MS system consisting of an Agilent 1290 Infinity Liquid Chromatography (LC) (Agilent, Santa Clara, CA, USA) coupled to an Agilent 6520 quadrupole time-of-flight mass spectrometer (QTOFMS) (Agilent). An Acquity T3 C18 column (Waters, Milford, MA, USA) was used for chromatographic separation. The mobile phases were 0.1% formic acid in water (A) and 0.1% formic acid in acetonitrile (B); LC gradient was run from 2% B to 90% B, 0–60 min; 90% B to 90% B, 60–70 min; 90% B to 2% B, 70–75 min; and 2% B to 2% B, 75–90 min. The LC-MS system was operated with an electrospray ionization (ESI) source; both positive ion and negative ion modes were performed. MS1 data were recorded for the mass range of 80–1000 at 1.03 scan/s, while MS/MS spectra of m/z 50–1000 collected at 1.4 scan/s. *QA/QC* details can be seen in the supplementary information.

### 4.4. Data Processing and Statistical Analysis

Raw data of MS1 acquired in Agilent d format were converted to mzdata using the MassHunter Workstation software (Agilent) and processed in XCMS (Scripps, La Jolla, CA, USA) [67] for peak picking, peak alignment and gap filling, etc. Then, two-tailed Welch’s *t*-test was performed to collect molecular features with group-differentiating peak intensity (*p* < 0.05, fold change >1.2). Tandem mass spectra of d format for features of interest were converted to abf using AnalysisBaseFile Converter (Riken, Japan) [68] and uploaded to MS-DIAL 2.90 (Riken, Japan) for a quality check before submission to compound identification. For multivariate analysis, master peak lists, including Crohn’s active vs. control and Crohn’s inactive vs. control, were first processed with normalization by sum and Pareto scaling before principal component analysis (PCA) in MetaboAnalyst 3.0 (Montreal, QC, Canada).

### 4.5. Compound Identification

Compound identification remains a bottleneck in metabolomics, challenging annotation coverage and biological interpretation. Here, we sought to apply a novel strategy that combines empirical MS/MS database search and in silico structure prediction for annotation and ranking. The analysis was implemented in MS-FINDER 2.30 (Riken, Japan), following a validated workflow [69,70]. Accurate mass, isotopic abundances, retention time, and MS/MS spectra were used to boost annotation confidence while reducing scoring bias. In MS-FINDER, both *Spectral database search* and *Formula prediction and structural elucidation* by in silico fragmentor were used. Scoring, through combining diagnostic metrics, were applied every step of the workflow. For database matching, we used *Precursor oriented spectral search* to query against major public MS/MS libraries, including MassBank (Tsuruoka, Yamagata, Japan), GNPS (La Jolla, San Diego, CA, USA), and ReSpect (Riken), etc. For in silico structure prediction, formula prediction and structural dereplication were performed. First, a list of formula candidates was generated based on accurate mass, neutral loss, and isotopic ratios, etc., using elements O, N, S, P, F, Cl, Br, and I. Then, the list was filtered according to Lewis/Senior rules, element probability, and element ratio at common range (99.7%). Top three formula candidates were used to retrieve all possible structures from 15 compound databases such as HMDB (Edmonton, AB, Canada) to compute in silico tandem mass spectra (at fragmentation tree depth of 2) for spectral analysis. The algorithm that underlies the virtual spectra prediction integrates bond energies, hydrogen-rearrangement, and heuristic rules.

### 4.6. Integrated Pathway Analysis

All the annotated metabolites, alongside their chemical identifiers, were uploaded to MetaboAnalyst 3.0 (Montreal, QC, Canada) [71] to identify perturbed pathways. The underlying algorithm consists of a hypergeometric test for over-representation analysis and relative-betweenness centrality for pathway topology analysis. Pathway results were shown in Figure 2. To better assess each individual pathway, a novel MetaMapp approach (Davis, CA, USA) [72] was used to visualize metabolic perturbations at the metabolite level. Eventually, the metabolites were mapped into integrated network clusters based on biochemical KEGG reactant pairs and/or chemical Tanimoto similarity scores, and details such as fold changes and *p*-values were shown for each individual metabolite.

## 5. Conclusions

LC-MS serum metabolomics was used to detect important metabolic changes and to discover novel compounds in an adult cohort with Crohn’s disease, covering both active and inactive cases. We resolve a distinct metabolic profile of Crohn’s disease from the control, with most metabolites downregulated in both active and inactive subjects. Metabolites identified embrace a diverse chemical space, pointing to important pathways perturbed in the Crohn’s disease, ranging from bioenergetic enhancement (e.g., β-oxidation of fatty acids) to altered signaling pathways (e.g., DHA cascades, tryptophan metabolism, etc.). Remarkably, the integral role of gut microbiota in mediating Crohn’s pathogenesis is highlighted; future studies that define and quantitate gut microbe’s etiologic contribution are crucial. Moreover, numerous xenobiotics and their biotransformants are detected, calling for massive exposomic profiling in future cohort studies as such. In summary, our study endorses using untargeted metabolomics for biomarker discovery, validation and etiologic inquiry; the findings may provide information and mechanistic insights needed for advancing current IBD research.

## Supplementary Materials

The following are available online at http://www.mdpi.com/1420-3049/24/3/449/s1. Figures S1 and S2; Tables S1–S3; *QA/QC* measures.
