# Supplementary material for: Serum Metabolomics Identifies Altered Bioenergetics, Signaling Cascades in Parallel with Exposome Markers in Crohn’s Disease
Source: Molecules. 2019 Jan 27;24(3):449. doi: 10.3390/molecules24030449 (PMC6385106; doi:10.3390/molecules24030449)
Supplement: Supplementary file 1 [file molecules-24-00449-s001.pdf]

Supplementary Information

Serum Metabolomics Identifies Altered Bioenergetics, Signaling Cascades in Parallel with Exposome Markers in Crohn's Disease

Yunjia Lai [1], Jingchuan Xue [1], Chih-Wei Liu [1], Bei Gao [2], Liang Chi [1], Pengcheng Tu [1], Kun Lu [1], Hongyu Ru [3]*

[1] Department of Environmental Sciences and Engineering, Gillings School of Global Public Health, CB #7431, University of North Carolina at Chapel Hill, NC 27599, United States; lai7@live.unc.edu (Y.L.); xuejc@email.unc.edu (J.X.); ericleah@email.unc.edu (C.L.); liang16@live.unc.edu (L.C.); ptu@live.unc.edu (P.T.); kunlu@unc.edu (K.L.).

[2] NIH West Coast Metabolomics Center, University of California at Davis, CA 95616, United States; beigao@ucdavis.edu (B.G.)

[3] Department of Population Health and Pathobiology, North Carolina State University, NC 27695, United States; hru@ncsu.edu (H.R.)

* Correspondence: hru@ncsu.edu; Tel.: +1 919-513-6915

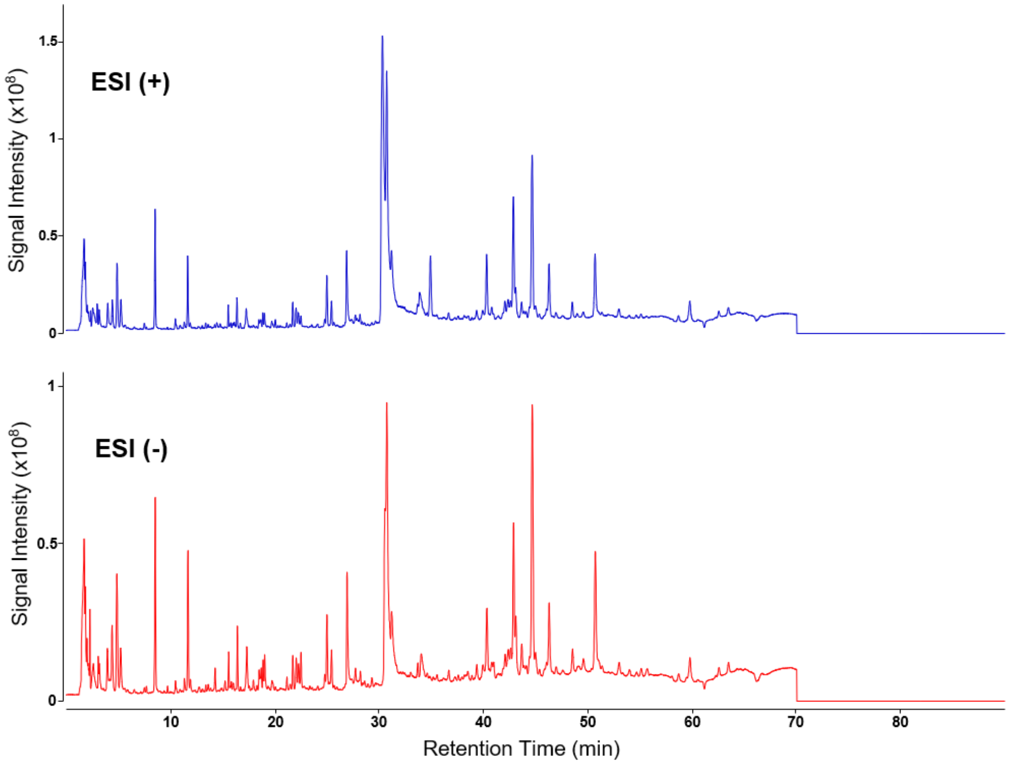

Figure S1. Representative total ion chromatograms for serum metabolites detected in ESI positive mode (upper panel) and ESI negative mode (lower panel) on LC-MS.

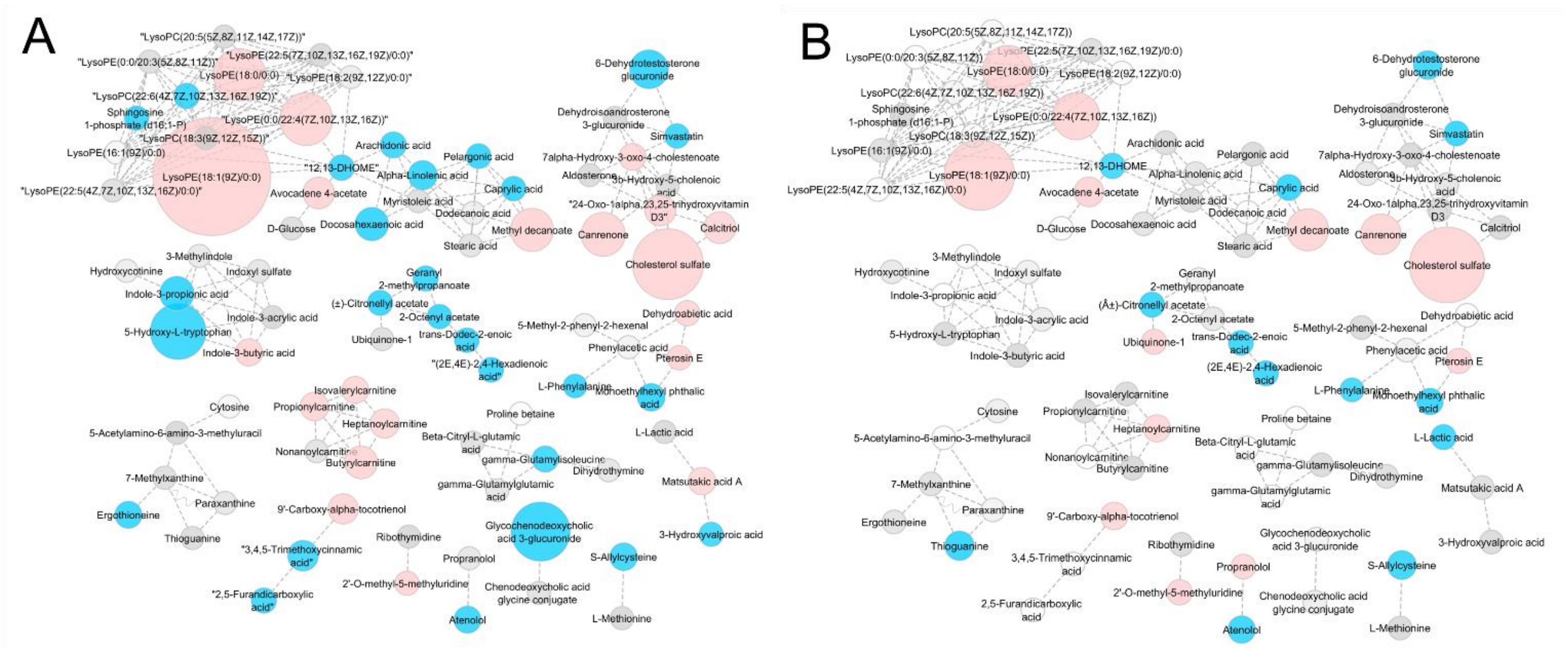

Figure S2. MetaMapp network visualization of metabolite-level changes in biochemical and/or chemical relationships, comparing inflammatory bowel disease versus control. Panel A gives metabolic changes for Crohn's active vs. Control, whereas Panel B Crohn's inactive vs. control. Here, one node represents one metabolite, with red and blue indicating upregulation and downregulation, respectively. Smaller *p*-values are indicated by lighter shades of color.

Table S1. List of compounds identified for the Crohn's cohorts. [1] the Level of Annotation (1-4) for each feature as outlined in Minimum Reporting Standards for Chemical Analysis by the Metabolomics Standards Initiative (MSI) [1, 2]. [2] "FC [a/c]" stands for fold change of active Crohn's as compared with Control; [3] "FC [a/i]" stands for fold change of active Crohn's vs. inactive Crohn's.

Compound Name	InChIKey	Level of Annotation [1]	FC [a/c] [2]	Change [a/c]	*p*-value [a/c]	FC [a/i] [3]	Change [a/i]	*p*-value [a/i]	ESI mode	Precursor Ion	Precursor *m/z*	RT (min)
2,3,5-Trimethoxy-6-(1E)-1-propenyl-2,5-cyclohexadiene-1,4-dione	RTBKABKNDSZTIA-AATRIKPKSA-N	1	2.01	Up	0.00000012	1.94	Up	0.00001349	pos	[M+H]+	239.0917	35.38
Monoethylhexyl phthalic acid	DJDSLBVSSOQSLW-UHFFFAOYNA-N	1	2.50	Down	0.00000238	1.89	Down	0.00002568	pos	[M+H]+	279.1594	55.22
Pterosin E	UYEZJDNVWNIIKS-UHFFFAOYNA-N	2	1.43	Up	0.00009140	1.47	Up	0.00015171	pos	[M+H]+	233.1187	39.31
Triisobutyl phosphate	HRKAMJBPFPHCSD-UHFFFAOYSA-N	1	2.34	Down	0.00023733	1.89	Down	0.00343767	pos	[M+H]+	267.1718	50.21
Phosphoric Acid Mono-[3-Amino-5-(5-Methyl-2,4-Dioxo-3,4-Dihydro-2h-Pyrimidin-1-Yl)-Tetrahydro-Furan-2-Ylmethyl] Ester	BQZMHQZNZNBJNF-GJMOJQLCSA-N	2	2.91	Down	0.00053955	1.61	Down	0.04989865	pos	[M+H]+	322.0794	3.95
Butyrylcarnitine	QWYFHHGCZUCMBN-UHFFFAOYNA-N	1	4.19	Up	0.00084045	2.03	Up	0.05344752	pos	[M+H]+	232.1540	35.07
Sphingosine 1-phosphate (d16:1-P)	IQHNJQKWEMCXAD-FOZJZALDNA-N	2	1.36	Down	0.00238964	1.23	Down	0.07546080	pos	[M+H]+	352.2232	38.15
(±)-Citronellyl acetate	JOZKFWLRHCDGJA-UHFFFAOYNA-N	1	1.74	Down	0.00316111	1.53	Down	0.00757509	pos	[M+H]+	199.1683	52.16
24-Oxo-1α,23,25-trihydroxyvitamin D3	ARRIBDAUGOLZSJ-LTFRBZSVNA-N	2	3.59	Up	0.00351258	1.79	Up	0.07694291	pos	[M+H]+	447.3085	42.80
6-Dehydrotestosterone glucuronide	GCIVSXAHMONYLO-HMAFJQTKSA-N	1	6.18	Down	0.00395415	2.61	Down	0.02871484	pos	[M+H]+	463.2328	41.12
2'-O-methyl-5-methyluridine	YHRRPHCORALGKQ-FDDDBJFASA-N	2	1.35	Up	0.00439937	1.48	Up	0.00004590	pos	[M+H]+	273.1089	39.29
Caprylic acid	WWZKQHOCKIZLMA-UHFFFAOYSA-N	1	1.53	Down	0.00516593	1.70	Down	0.00081482	pos	[M+H]+	145.1223	39.82
Glycochenodeoxycholic acid 3-glucuronide	ABFZMYIIUREPLL-ZWDGUQLWSA-N	1	12.95	Down	0.00526718	1.30	Down	0.47669904	pos	[M+H]+	626.3526	35.05

L-Phenylalanine	COLNVLDHVKWLRT-QMMMGPOBSA-N	1	1.21	Down	0.00531461	1.13	Down	0.04708596	pos	[M+H]+	166.0862	11.09
Indole-3-propionic acid	GOLXRNDWAUTYKT-UHFFFAOYSA-N	1	4.69	Down	0.00569155	1.19	Down	0.69102692	pos	[M+H]+	190.0852	30.81
9'-Carboxy-α-tocotrienol	MEZYOZBFEXYIKB-ASKDMKCJSA-N	1	3.29	Up	0.00784716	2.17	Up	0.04152933	pos	[M+H]+	387.2510	38.71
Indole-3-butyric acid	JTEDVYBZBROSJT-UHFFFAOYSA-N	1	2.33	Up	0.00829724	2.31	Up	0.08057940	pos	[M+H]+	204.1029	23.27
(2E,4E)-2,4-Hexadienoic acid	WSWCOQWTEOXDQX-MQQKCMAXSA-N	1	1.63	Down	0.00868860	1.47	Down	0.02560559	pos	[M+H]+	113.0601	17.58
Pelargonic acid	FBUKVWPVBMHYJY-UHFFFAOYSA-N	1	1.63	Down	0.01023227	1.45	Down	0.06353545	pos	[M+H]+	159.1381	25.64
2-Octenyl acetate	MBRLTLPMVMFRTJ-FPLPWBNLSA-N	1	1.50	Down	0.01104562	1.23	Down	0.28585419	pos	[M+H]+	171.1380	32.32
Ergothioneine	SSISHJJTAXXQAX-ZETCQYMHSA-N	1	2.31	Down	0.01200790	1.32	Down	0.24507362	pos	[M+H]+	230.0960	2.21
α-Linolenic acid	DTOSIQBPPRVQHS-PDBXOOCHSA-N	1	3.02	Down	0.01393572	1.49	Down	0.25963934	pos	[M+H]+	279.2310	61.00
LSM-15135	PJAYKLYFYLNPHZ-KAIUQPJCNA-N	1	1.64	Down	0.01435329	1.09	Down	0.61822021	pos	[M+H]+	467.2613	46.11
LysoPC(22:6(4Z,7Z,10Z,13Z,16Z,19Z))	LSOWKZULVQWMLY-APPDJCNMSA-N	1	1.81	Down	0.01453815	1.11	Up	0.52934087	pos	[M+H]+	568.3380	47.24
(3E,5Z)-Tetradecadienoate	YRUMHTHCEZRHTN-XAZJVICWSA-M	2	1.42	Down	0.01655718	1.26	Down	0.03600126	pos	[M+H]+	225.1835	51.39
Geranyl 2-methylpropanoate	OGJYXQFXLSCKTP-LCYFTJDESA-N	1	1.70	Down	0.01870596	1.18	Down	0.43207215	pos	[M+H]+	225.1844	64.36
Simvastatin	RYMZZMVNJRMUDD-HGQWONQESA-N	1	1.45	Down	0.01904638	1.35	Down	0.00083845	pos	[M+H]+	419.2771	56.35
Parthenolide	KTEXNACQROZXEV-RXBUGHCGNA-N	2	2.69	Down	0.01972979	1.69	Down	0.17932579	pos	[M+H]+	249.1465	17.45
5-Hydroxy-L-tryptophan	LDCYZAJDBXYCGN-UHFFFAOYNA-N	1	11.85	Down	0.02005146	3.63	Down	0.07577307	pos	[M+H]+	221.0933	7.05
7α-Hydroxy-3-oxo-4-cholestenoate	SATGKQGFUDXGAX-QLHYZZJLNA-N	1	2.15	Up	0.02126665	1.50	Up	0.06262454	pos	[M+H]+	431.3156	51.03

Arachidonic acid	YZXBAPSDXZZRGB-DOFZRALJSA-N	1	2.05	Down	0.02137707	1.50	Down	0.12431059	pos	[M+H]+	305.2469	63.76
Dehydroabietic acid	NFWKVWVWBFBAOV-IPNZSQQUSA-N	1	1.68	Up	0.02223098	1.01	Down	0.93759620	pos	[M+H]+	301.2179	26.60
Calcitriol	GMRQFYUYWCNGIN-NKMMMXOESA-N	1	3.91	Up	0.02238014	1.59	Up	0.05877359	pos	[M+H]+	417.3355	52.91
Atenolol	METKIMKYRPQLGS-UHFFFAOYSA-N	1	2.34	Down	0.02318934	2.15	Down	0.01960119	pos	[M+H]+	267.1716	50.14
12,13-DHOME	CQSLTKIXAJTQGA-FLIBITNWNA-N	1	1.76	Down	0.02513131	1.61	Down	0.04717503	pos	[M+H]+	315.2527	65.90
trans-Dodec-2-enoic acid	PAWGRNGPMLVJQH-ZHACJKMWSA-N	1	1.46	Down	0.02518177	1.38	Down	0.02369593	pos	[M+H]+	199.1688	59.53
Docosahexaenoic acid	MBMBGCFOFBJSGT-KUBAVDMBSA-N	1	4.17	Down	0.02536038	1.67	Down	0.23455029	pos	[M+H]+	329.2477	62.97
Propionylcarnitine	UFAHZIUFPNSHSL-UHFFFAOYNA-N	1	2.80	Up	0.02630172	1.86	Up	0.10660293	pos	[M+H]+	218.1396	32.66
S-Allylcysteine	ZFAHNWWNDFHPOH-UHFFFAOYNA-N	1	2.52	Down	0.02883093	2.66	Down	0.02462844	pos	[M+H]+	162.0582	6.71
gamma-Glutamylisoleucine	SNCKGJWJABDZHI-WHOSYVTDNA-N	1	1.96	Down	0.03257083	1.67	Down	0.08462893	pos	[M+H]+	261.1445	15.87
2,5-Furandicarboxylic acid	CHTHALBTIRVDBM-UHFFFAOYSA-N	2	1.65	Down	0.03372017	1.06	Down	0.72365954	pos	[M+H]+	157.0142	4.33
Heptanoylcarnitine	VDPCTFWULDLKHT-UHFFFAOYNA-N	2	3.01	Up	0.03514665	2.13	Up	0.04767880	pos	[M+H]+	274.1996	47.00
Matsutakic acid A	VSKXYKGCLVJSEW-UHFFFAOYNA-N	1	2.43	Up	0.03516141	1.65	Up	0.15544906	pos	[M+H]+	201.1122	32.59
Isovalerylcarnitine	IGQBPDJNUXPEMT-UHFFFAOYNA-N	1	2.24	Up	0.03606865	1.61	Up	0.09112958	pos	[M+H]+	246.1685	38.90
3-Hydroxyvalproic acid	LLPFTSMZBSRZDV-UHFFFAOYNA-N	1	1.39	Down	0.04358658	1.41	Down	0.09670605	pos	[M+H]+	161.1161	13.96
3,4,5-Trimethoxycinnamic acid	YTFVRYKNXDADBI-SNAWJCMRSA-N	1	3.47	Down	0.04541668	1.10	Up	0.84296594	pos	[M+H]+	239.0914	24.15
5-Acetylamino-6-amino-3-methyluracil	POQOTWQIYYNXAT-UHFFFAOYSA-N	2	1.91	Up	0.05314924	1.18	Down	0.68391172	pos	[M+H]+	199.0808	7.63

Indole-3-acrylic acid	PLVPPLCLBIEYEA-WAYWQWQTSA-N	1	1.22	Down	0.05771250	1.09	Down	0.36774566	pos	[M+H]+	188.0698	15.58
Ribothymidine	DWRXFEITVBNRMK-JXOAFFINSA-N	1	1.75	Down	0.06020041	1.62	Down	0.10530982	pos	[M+H]+	259.0942	4.23
LysoPE(22:5(7Z,10Z,13Z,16Z,19Z)/0:0)	OSRWIBSZJTWIKA-SCFYABBUSA-N	2	1.74	Up	0.06105994	1.40	Up	0.16189187	pos	[M+H]+	528.3073	49.68
Dehydroisoandrosterone 3-glucuronide	GLONBVCUAVPJFV-CLIRWHJISA-N	1	1.86	Up	0.06292079	1.71	Up	0.12284762	pos	[M+H]+	465.2484	56.10
L-Methionine	FFEARJCKVFRZRR-BYPYZUCNSA-N	1	1.47	Down	0.06777699	1.43	Down	0.06416236	pos	[M+H]+	150.0577	3.30
LysoPC(18:3(9Z,12Z,15Z))	WKQNRCYKYCKESD-YVHLTTHBSA-N	1	1.49	Up	0.07746452	1.08	Down	0.72753743	pos	[M+H]+	518.3259	44.74
Aldosterone	PQSUYGKTWSAVDQ-SRPWZAMTSA-N	1	1.39	Down	0.08182447	1.15	Up	0.32676505	pos	[M+H]+	361.2023	30.55
3b-Hydroxy-5-cholenoic acid	HIAJCGFYHIANNA-HPIXXDBXNA-N	1	4.22	Down	0.08876483	2.71	Up	0.24520638	pos	[M+H]+	375.2873	45.55
L-Lactic acid	JVTAAEKCZFNVCJ-RUOZJJLTNA-N	1	1.35	Down	0.09066944	1.48	Down	0.01574859	pos	[M+H]+	91.0397	3.35
LysoPC(20:5(5Z,8Z,11Z,14Z,17Z))	PDIGSOAOQOXRDU-WJPZTBRDSA-N	1	1.72	Down	0.09558370	1.19	Down	0.57586369	pos	[M+H]+	542.3237	44.46
Ulipristal acetate	OOLLAFOLCSJHRE-ZHAKMVSLSA-N	2	1.62	Up	0.10617879	1.12	Down	0.56839007	pos	[M+H]+	476.2826	44.15
Thioguanine	WYWHKKSPHMUBEB-UHFFFAOYSA-N	2	1.38	Down	0.10676364	2.80	Down	0.00034464	pos	[M+H]+	168.0346	30.30
LysoPC(18:3(6Z,9Z,12Z))	MRTUWVDDQVMUCR-ACHCNROVSA-N	1	1.19	Down	0.11792120	1.07	Down	0.13069027	pos	[M+H]+	518.3233	48.83
D-Glucose	WQZGKKKJIJFFOK-GASJEMHNSA-N	1	1.47	Up	0.14023224	1.16	Down	0.66628804	pos	[M+H]+	181.0712	14.18
7-Methylxanthine	PFWLFWPASULGAN-UHFFFAOYSA-N	1	2.53	Down	0.14103080	2.40	Down	0.18589556	pos	[M+H]+	167.0562	12.98
LysoPE(0:0/20:3(5Z,8Z,11Z))	FJDVENKXPUIXRG-WMTBOZPISA-N	1	1.58	Up	0.14721517	1.01	Down	0.97617820	pos	[M+H]+	504.3060	48.74
Myristoleic acid	YWWVWXASSLXJHU-WAYWQWQTSA-N	1	1.65	Up	0.15060851	2.36	Up	0.12243280	pos	[M+H]+	227.1994	45.40

Ubiquinone-1	SOECUQMRSRVZQQ-UHFFFAOYSA-N	1	1.22	Up	0.15804160	1.36	Up	0.00035947	pos	[M+H]+	251.1263	45.40
LysoPE(22:5(4Z,7Z,10Z,13Z,16Z)/0:0)	GUWNUQBHHWXHBE-GOGKNFEWSA-N	2	1.50	Up	0.15936736	1.09	Up	0.75055930	pos	[M+H]+	528.3086	48.30
β-Citryl-L-glutamic acid	GAQNUGISBQJMKO-UHFFFAOYNA-N	2	1.24	Down	0.16591115	1.03	Down	0.83778341	pos	[M+H]+	322.0766	6.82
5'-Methylthioadenosine	WUUGFSXJNOTRMR-IOSLPCCCSA-N	1	1.18	Up	0.16961838	1.20	Up	0.15108435	pos	[M+H]+	298.0985	15.21
Chenodeoxycholic acid glycine conjugate	GHCZAUBVMUEKKP-YDVLHWCRNA-N	1	26.56	Down	0.19209194	2.61	Down	0.40614603	pos	[M+H]+	450.3220	39.48
Nonanoylcarnitine	MPSPNFAQQQMFLK-UHFFFAOYNA-N	1	1.51	Up	0.22273708	1.14	Down	0.74371959	pos	[M+H]+	302.2303	28.80
Stearic acid	QIQXTHQIDYTFRH-UHFFFAOYSA-N	1	1.36	Up	0.22840365	1.41	Up	0.10401974	pos	[M+H]+	285.2781	70.69
D,L-Cyclohexanephosphinothricin	ZAIBDODFHKWMIG-UHFFFAOYNA-M	2	1.19	Up	0.25278998	1.36	Up	0.07149174	pos	[M+H]+	222.0894	49.63
γ-Glutamylglutamic acid	OWQDWQKWSLFFFR-IOMOGOHMNA-N	1	1.53	Down	0.25986788	1.11	Down	0.82237316	pos	[M+H]+	277.1021	2.88
Indoxyl sulfate	BXFFHSIDQOFMLE-UHFFFAOYSA-N	2	1.61	Down	0.26222779	1.45	Up	0.43719360	pos	[M+H]+	214.0155	22.16
Propranolol	AQHHHDLHHXJYJD-UHFFFAOYSA-N	2	1.23	Up	0.27184370	1.32	Up	0.02484055	pos	[M+H]+	260.1664	46.13
Dihydrothymine	NBAKTGXDIBVZOO-UHFFFAOYNA-N	1	1.44	Up	0.27809666	1.66	Down	0.15573618	pos	[M+H]+	129.0659	1.95
Methyl cinnamate	CCRCUPLGCSFEDV-FPLPWBNLSA-N	1	1.18	Up	0.27841737	1.29	Up	0.00253455	pos	[M+H]+	163.0756	42.43
Paraxanthine	QUNWUDVFRNGTCO-UHFFFAOYSA-N	1	1.87	Down	0.28646028	1.60	Down	0.46275957	pos	[M+H]+	181.0718	15.74
LysoPC(20:4(8Z,11Z,14Z,17Z))	GOMVPVRDBLLHQC-VEJNOCSESA-N	1	1.19	Down	0.30754597	1.17	Down	0.31391066	pos	[M+H]+	544.3402	46.31
Corallopyronin A	FPBHSTHTCPCNBS-KMCNCHRSNA-N	2	1.57	Down	0.31903603	1.22	Up	0.51337778	pos	[M+H]+	528.2994	47.01
2-Aminoheptanoate	RDFMDVXONNIGBC-UHFFFAOYNA-N	1	1.12	Down	0.37384841	1.03	Up	0.80661879	pos	[M+H]+	146.1176	2.09

LysoPE(18:2(9Z,12Z)/0:0)	DBHKHNGBVGWQJE-USWSLJGRSA-N	1	1.28	Up	0.37511829	1.12	Down	0.63061274	pos	[M+H]+	478.2921	46.67
Hydroxycotinine	XOKCJXZZNAUIQN-IENPIDJESA-N	1	2.05	Up	0.37944805	13.73	Down	0.30149033	pos	[M+H]+	193.0981	2.98
Senecioic acid	YYPNJNDODFVZLE-UHFFFAOYSA-N	1	1.16	Down	0.39043044	1.21	Down	0.21185416	pos	[M+H]+	101.0598	11.53
(9S,10S)-9,10-dihydroxyoctadecanoate	VACHUYIREGFMSP-RXQGYGPJNA-N	1	1.13	Down	0.41123009	1.15	Down	0.09478146	pos	[M+H]+	317.2684	60.31
LysoPC(20:4(5Z,8Z,11Z,14Z))	LAXQYRRMGGEGOH-JXRLJXCWSA-N	1	1.17	Down	0.42149622	1.16	Down	0.46194975	pos	[M+H]+	544.3406	46.39
Phenylacetic acid	WLJVXDMOQOGPHL-UHFFFAOYSA-N	1	2.02	Up	0.46162512	1.34	Up	0.43715042	pos	[M+H]+	137.0603	30.58
3-Hydroxysuberic acid	ARJZZFJXSNJKGR-UHFFFAOYNA-N	2	1.13	Down	0.46183185	1.19	Down	0.34598512	pos	[M+H]+	191.0901	17.66
5-Methyl-2-phenyl-2-hexenal	YURDCJXYOLERLO-LCYFTJDESA-N	2	1.23	Down	0.46522402	1.48	Down	0.11108539	pos	[M+H]+	189.1260	8.44
cis-Aconitic acid	GTZCVFVGUGFEME-IWQZZHSRSA-N	1	1.14	Down	0.51428003	1.37	Up	0.11241819	pos	[M+H]+	175.0234	4.92
LysoPE(16:1(9Z)/0:0)	DSOWUEHXZJUNID-WHXUGTBJSA-N	1	1.25	Up	0.54487432	1.74	Down	0.17590534	pos	[M+H]+	452.2778	44.78
Cytosine	OPTASPLRGRRNAP-UHFFFAOYSA-N	2	1.36	Up	0.54810195	1.56	Down	0.39410340	pos	[M+H]+	112.0495	6.21
Persin	IBDVBNUJEGRVQN-NQLNTKRDNA-N	1	1.16	Up	0.57078057	1.31	Up	0.39578507	pos	[M+H]+	381.2979	69.16
Proline betaine	CMUNUTVVOOHQPW-LURJTMIESA-N	1	1.26	Up	0.63922805	1.29	Down	0.68321720	pos	[M+H]+	144.1018	2.15
3-Hydroxydodecanedioic acid	FYVQCLGZFXHEGL-UHFFFAOYNA-N	1	1.09	Up	0.64697586	1.11	Up	0.60691447	pos	[M+H]+	247.1534	29.05
D-Limonene	XMGQYMWWDOXHJM-SNVBAGLBSA-N	1	1.09	Up	0.68927767	1.40	Up	0.09540662	pos	[M+H]+	137.1325	63.69
Palmitic acid	IPCSVZSSVZVIGE-UHFFFAOYSA-N	1	1.07	Down	0.73279017	1.08	Up	0.64989658	pos	[M+H]+	257.2473	63.69
Leucylproline	VTJUNIYRYIAIHF-UHFFFAOYNA-N	1	1.06	Up	0.73624653	1.03	Down	0.80241990	pos	[M+H]+	229.1546	3.48

L-Valine	KZSNJWFQEVHDMF-SGAVLPGINA-N	1	1.03	Down	0.82807666	1.09	Down	0.59022497	pos	[M+H]+	118.0871	2.30
MG(16:0/0:0/0:0)	QHZLMUACJMDIAE-SFHVURJKSA-N	1	1.05	Down	0.82849371	1.10	Up	0.63294725	pos	[M+H]+	331.2841	63.69
Cinnamic acid	WBYWAXJHAXSJNI-SREVYHEPSA-N	1	1.01	Down	0.85359641	1.06	Down	0.22051079	pos	[M+H]+	149.0589	37.43
Suberic acid	TYFQFVWCELRYAO-UHFFFAOYSA-N	1	1.03	Up	0.87277712	1.09	Down	0.55798882	pos	[M+H]+	175.0963	22.77
Acetylisoniazid	CVBGNAKQQUWBQV-UHFFFAOYSA-N	2	1.09	Down	0.87761371	1.79	Down	0.30178412	pos	[M+H]+	180.0779	3.18
(2R)-3-(Phosphonooxy)propane-1,2-diyl diheptanoate	JAXUAGQDLYDLQB-GGYSOQFKNA-N	2	1.03	Down	0.88393070	1.06	Up	0.74717121	pos	[M+H]+	397.1967	67.63
Pipecolic acid	HXEACLLIILLPRG-UHFFFAOYNA-N	1	1.03	Down	0.89560337	1.70	Up	0.21050538	pos	[M+H]+	130.0861	2.99
Veratrosine	WXQHVBNTINGJJR-ZAFUCLPBNA-N	2	1.01	Up	0.94704686	1.11	Up	0.67099596	pos	[M+H]+	572.3614	48.52
3-Hydroxy-9-hexadecenoylcarnitine	WAGYLURELCUJPG-RPZXQYRRSA-N	2	1.01	Up	0.94961092	1.04	Up	0.58115810	pos	[M+H]+	414.3214	56.44
3-Cyclohexyl-1,1-bis(2-methylpropyl)thiourea	RQQYMIYZTDXXRP-UHFFFAOYSA-N	2	1.03	Up	0.95041497	1.50	Up	0.26507870	pos	[M+H]+	271.2201	63.78
PE(18:1(9Z)/20:4(5Z,8Z,11Z,14Z))	PECSWFQRRFRZPW-BHPGJWMBSA-N	2	1.01	Down	0.96190425	1.07	Down	0.58985875	pos	[M+H]+	766.5307	54.59
Heptaethylene glycol	XPJRQAIZZQMSCM-UHFFFAOYSA-N	2	1.00	Down	0.99950523	1.11	Down	0.62838470	pos	[M+H]+	327.2014	17.97
3-Methylindole	ZFRKQXVRDFCRJG-UHFFFAOYSA-N	2	2.22	Up	0.32027247	1.22	Up	0.17802053	neg	[M-H]-	130.0674	12.66
Avocadene 4-acetate	MFLWBVVCOWPUBA-UHFFFAOYNA-N	2	3.85	Up	0.00000962	1.23	Up	0.25921122	neg	[M-H]-	327.2556	40.89
Canrenone	UJVLDDZCTMKXJK-WNHSNXHDSA-N	2	7.88	Up	0.00000000	1.20	Up	0.18696924	neg	[M-H]-	339.1997	39.91
Caprylic acid	WWZKQHOCKIZLMA-UHFFFAOYSA-N	1	2.88	Up	0.00032049	1.03	Up	0.89523757	neg	[M-H]-	143.1070	19.41
Cholesterol sulfate	BHYOQNUELFTYRT-ZMWIUTBPNA-N	2	16.88	Up	0.00006077	1.16	Down	0.55328249	neg	[M-H]-	465.3039	42.56

Docosahexaenoic acid	MBMBGCFOFBJSGT-KUBAVDMBSA-N	1	4.41	Down	0.01025668	2.98	Down	0.01329273	neg	[M-H]-	327.2346	46.33
Dodecanoic acid	POULHZVOKOAJMA-UHFFFAOYSA-N	1	1.24	Down	0.40349789	1.06	Down	0.85001991	neg	[M-H]-	199.1695	37.98
LysoPE(0:0/22:4(7Z,10Z,13Z,16Z))	VLFJNHPNIOTOOA-YMIVKDKMSA-N	2	10.93	Up	0.00000027	1.23	Up	0.22413570	neg	[M-H]-	528.3093	35.60
LysoPE(18:0/0:0)	BBYWOYAFBUOUFP-JOCHJYFZSA-N	1	10.67	Up	0.00000087	1.09	Up	0.62142070	neg	[M-H]-	480.3100	37.10
LysoPE(18:1(9Z)/0:0)	PYVRVRFVLRNJLY-MZMPXXGTSA-N	1	32.73	Up	0.00000746	1.93	Up	0.01602767	neg	[M-H]-	478.2931	37.54
LysoPE(18:2(9Z,12Z)/0:0)	DBHKHNGBVGWQJE-USWSLJGRSA-N	1	148.04	Down	0.00041488	2.16	Down	0.42241726	neg	[M-H]-	476.2814	35.10
Methyl decanoate	YRHYCMZPEVDGFQ-UHFFFAOYSA-N	2	7.56	Up	0.00000256	1.12	Up	0.35145249	neg	[M-H]-	185.1531	33.03
Perillic acid	CDSMSBUVCWHORP-UHFFFAOYNA-N	1	1.20	Down	0.58934215	1.85	Down	0.00569642	neg	[M-H]-	165.0919	21.46
Phomopsichalasin	AEKBQYOSHUYACR-HUBYWGRONA-N	2	1223.64	Up	0.00026964	4.64	Up	0.00528030	neg	[M-H]-	502.2980	36.61

Table S2. List of metabolic pathways as matched in MetaboAnalyst 3.0.

Compound name	# Hit of metabolites	-LOG(p)	Impact
Caffeine metabolism	3	5.312	0.27813
Fatty acid biosynthesis	3	3.0119	
Glycolysis or Gluconeogenesis	2	2.3377	
Phenylalanine metabolism	2	1.728	0.17318
Primary bile acid biosynthesis	2	1.6607	0.01029
Starch and sucrose metabolism	2	1.5665	0.02968
Linoleic acid metabolism	1	1.4794	
Pyrimidine metabolism	2	1.3005	0.03619
Sphingolipid metabolism	1	1.0481	0.1402
Aminoacyl-tRNA biosynthesis	2	1.001	
Phenylalanine, tyrosine and tryptophan biosynthesis	1	0.98684	0.00062
alpha-Linolenic acid metabolism	1	0.93094	0.20335
Pentose phosphate pathway	1	0.85568	
Pyruvate metabolism	1	0.85568	0.13756
Propanoate metabolism	1	0.78905	
Nitrogen metabolism	1	0.71119	
Glycerophospholipid metabolism	1	0.71119	0.00317
Galactose metabolism	1	0.67622	0.00276
Steroid hormone biosynthesis	2	0.67576	0.01235
Histidine metabolism	1	0.628	0.00861
Pentose and glucuronate interconversions	1	0.50794	0.009
Cysteine and methionine metabolism	1	0.47451	0.03806
Arachidonic acid metabolism	1	0.4154	0.21669
Drug metabolism - cytochrome P450	1	0.2512	0.0044
Amino sugar and nucleotide sugar metabolism	1	0.24125	0.27813

Table S3. Summary of the clinical data of enrolled patients and controls.

Subject ID	Disease	Active / Inactive	Score	Gender	Age at Blood Draw	Age at Diagnosis	Disease Duration (yrs)
113	CD	Active	17	F	66	18	48
114	CD	Active	10	M	79	33	46
115	CD	Active	10	M	55	32	23
129	CD	Active	11	F	62	35	27
139	CD	Active	14	F	42	17	25
148	CD	Active	10	F	32	25	7
156	CD	Active	13	M	43	32	11
157	CD	Active	11	M	29	15	14
158	CD	Active	12	F	24	21	3
159	CD	Active	21	M	57	19	38
141	CD	Inactive	0	M	22	14	8
143	CD	Inactive	0	F	46	45	1
145	CD	Inactive	0	F	28	15	13
146	CD	Inactive	0	M	60	26	34
147	CD	Inactive	0	M	32	21	11
149	CD	Inactive	0	M	71	41	30
150	CD	Inactive	0	F	60	27	33
151	CD	Inactive	0	F	24	10	14
152	CD	Inactive	0	F	25	18	7
153	CD	Inactive	0	M	27	24	3
104	HC	n/a	n/a	F	59	n/a	n/a
106	HC	n/a	n/a	F	60	n/a	n/a
107	HC	n/a	n/a	F	53	n/a	n/a
110	HC	n/a	n/a	M	61	n/a	n/a
111	HC	n/a	n/a	M	53	n/a	n/a
116	HC	n/a	n/a	M	23	n/a	n/a
117	HC	n/a	n/a	M	40	n/a	n/a
118	HC	n/a	n/a	M	51	n/a	n/a
119	HC	n/a	n/a	F	69	n/a	n/a
120	HC	n/a	n/a	F	52	n/a	n/a

QA/QC Procedures of Metabolomic Analysis

QA/QC metabolomics practice include real-time mass calibration, intermittent injection of QC (pooled extracts), sample randomization and blank injection, which has been strictly applied to the present study. Three no-injections, one solvent blank injection, and one QC sample injection were performed before the analysis of the bulk test samples. QC samples were analyzed every 8 samples to evaluate potential batch effects. As validated, the whole procedure ensures that (1) chromatographic conditions are sufficiently equilibrated, (2) stable back pressure reached, and (3) batch effect monitoring covers all test samples.

References

1. Fiehn O, Robertson D, Griffin J, van der Werf M, Nikolau B, Morrison N, Sumner LW, Goodacre R, Hardy NW, Taylor C *et al*: **The metabolomics standards initiative (MSI)**. *Metabolomics* 2007, **3**(3):175-178.
2. Blaženović I, Kind T, Ji J, Fiehn O: **Software Tools and Approaches for Compound Identification of LC-MS/MS Data in Metabolomics**. *Metabolites* 2018, **8**(2):31.
